# Gut Microbiota as a Key Modulator of Chronic Disease: Implications for Diabetes, Autoimmunity, and Cancer

**DOI:** 10.7759/cureus.84687

**Published:** 2025-05-23

**Authors:** Noor Inayat, Asma Zahir, Ahmad J Hashmat, Anusha Khan, Aftab Ahmad, Meenal Sikander, Shayan Zakir, Sabeena Ahmad, Saira K Awan, Syed S Raza, Giustino Varrassi

**Affiliations:** 1 Internal Medicine, Khyber Medical University Institute of Medical Sciences, Kohat, PAK; 2 Internal Medicine, Hayatabad Medical Complex Peshawar, Peshawar, PAK; 3 Internal Medicine, Richmond University Medical Center, Staten Island, USA; 4 Medical Ethics, Northwell Health, Long Island Jewish Medical Center, New Hyde Park, USA; 5 General Medicine, Cork University Hospital, Cork, IRL; 6 Medicine, Rehman Medical Institute, Peshawar, PAK; 7 Anesthesiology, University of Virginia Medical Center, Charlottesville, USA; 8 Oncology/Hematology, University of Virginia Medical Center, Charlottesville, USA; 9 Neurology, Advanced Neurology P.C., Brooklyn, USA; 10 Medicine, Kabir Medical College, Peshawar, PAK; 11 Physiology, Khyber Medical College/Khyber Teaching Hospital, Peshawar, PAK; 12 Internal Medicine, Community Medical Center, Fresno, USA; 13 Pain Medicine, Fondazione Paolo Procacci, Rome, ITA

**Keywords:** autoimmunity, cancer, endocrinology and diabetes, gut-brain axis, gut microbiota dysbiosis

## Abstract

The gut microbiota (GM) represents an intricate, dynamic, and complex ecosystem. It plays a key role in health and disease. The GM interacts with the host and modulates various physiological functions, including metabolism, immune regulation, and neurological function. This narrative review comprehensively analyses the role of the GM in the development and progression of three major chronic conditions, namely diabetes, autoimmune disorders, and cancer. Using a structured literature search strategy across databases such as Google Scholar, PubMed, Scopus, and Web of Science, relevant studies published between 2000 and 2025 were identified and analysed. This review highlights that dysbiosis contributes significantly to the pathogenesis of these chronic conditions. In type 2 diabetes mellitus (T2DM), alterations in the GM are associated with systemic inflammation, insulin resistance, and decreased microbial diversity. Similarly, in autoimmune disorders such as rheumatoid arthritis (RA), multiple sclerosis (MS), and inflammatory bowel disease (IBD), dysbiosis disrupts immune homeostasis, which in turn causes sustained inflammation and aberrant immune responses. Lastly, dysbiosis has been linked to the onset and progression of various gastrointestinal cancers through mechanisms including chronic inflammation and the production of carcinogenic metabolites. Fecal microbiota transplantation (FMT), probiotics, prebiotics, and dietary modifications are being explored for their potential to restore microbial balance and improve clinical outcomes. In conclusion, this review highlights the GM’s pivotal role in the pathogenesis of chronic diseases and its potential as a therapeutic target.

## Introduction and background

The human body harbors a vast and intricate microbial ecosystem. The gut microbiota (GM) plays a vital role in both health and disease. Trillions of microorganisms constitute the GM. These include archaea, bacteria, viruses, and fungi. The GM interacts with the host and modulates various physiological functions, including metabolism, immune regulation, and neurological function [1,2]. In recent times, research has linked the GM as a key modulator of chronic diseases, especially in conditions such as diabetes, autoimmune disorders, and cancer [2,3]. These conditions were once thought to be primarily influenced by genetic and environmental factors. They are now believed to be molded by the composition and function of the GM.

Microbes are essential for maintaining gut homeostasis and influencing multiple mechanisms. These include fermentation of dietary fibers, maintaining the integrity of the intestinal barrier, inflammatory pathway modulation, and short-chain fatty acids (SCFAs) production [4]. Dysbiosis is an imbalance in the composition and function of the microbiota. It is involved in the pathogenesis of several chronic diseases. For instance, in type 2 diabetes mellitus (T2DM), alterations in the microbial composition and reduction in beneficial bacteria have been associated with systemic inflammation and insulin resistance [5].

Likewise, in autoimmune conditions such as rheumatoid arthritis (RA), inflammatory bowel disease (IBD), and multiple sclerosis (MS), disruptions in gut microbial homeostasis can elicit irregular immune responses. This can lead to sustained inflammation and tissue damage [6,7]. Furthermore, the role of the microbiota in causing cancer has, in recent times, become a topic of great interest. Emerging evidence has suggested that microbial metabolites and inflammatory pathways can stimulate or impede tumor development in various cancers. Research has found that certain bacterial species, such as *Fusobacterium nucleatum*, have been implicated in the development of colorectal cancer, thus suggesting that targeting the gut microbiome could be a viable approach in both cancer prevention and therapy [8,9].

Despite these developments and a rapidly growing body of evidence, the exact mechanisms by which the GM influences chronic diseases still remain incompletely understood. Antibiotics, diet, genetics, and lifestyle contribute to the dynamic nature of the gut microbiome. Which makes it highly variable among individuals [5].

Associations between gut dysbiosis and disease states have been well documented; however, differentiating causation from correlation remains a major challenge. It is noteworthy that understanding the interaction between host physiology with specific microbes and their metabolites could pave the way for novel therapeutic interventions. These include fecal microbiota transplantation (FMT), probiotics, prebiotics, and microbiome-targeted drug development [1,7].

This review investigates the pivotal role of the gut microbiota in the development of three major chronic medical conditions: diabetes, autoimmune disorders, and cancer. We will explore the mechanisms by which gut microbes influence disease progression. Recent advancements in microbiome research will also be discussed. Analysing the complex relationship between the GM and chronic diseases in this study, we aim to provide a comprehensive understanding of the subject and its potential as a therapeutic and preventive agent in modern medicine.

## Review

Methodology

This narrative review employed a comprehensive approach to gather, evaluate, and synthesize relevant literature.

Literature Search Strategy

A comprehensive literature search was conducted to narratively review the role of the gut microbiota in chronic diseases such as diabetes, autoimmune disorders, and cancer. Multiple databases, including Google Scholar, PubMed, Web of Science, and Scopus, were used. The search terms were constructed using a combination of keywords and medical subject headings (MeSH) terms. The search was based on the following key terms combined with Boolean operators (AND, OR): "Chronic disease OR diabetes OR autoimmune diseases OR cancer", "Gut microbiota OR microbiome", "Dysbiosis OR microbial imbalance", "Type 2 diabetes OR insulin resistance".

Inclusion Criteria

To ensure that the latest literature is gathered, the search was limited to studies published between 2000 and 2025. Data from original research articles, systematic reviews, meta-analyses, and clinical trials was included. Studies published only in English were selected. Studies involving human participants, animal models, or in vitro experiments that focused on the subject under consideration were included. 

Exclusion Criteria

Non-English publications and conference abstracts were excluded due to language barriers and limited data in these formats. Furthermore, studies focused on acute diseases or non-gut-related microbial impacts were also excluded. Articles unrelated to the gut microbiota, such as those on oral microbiota or microbiota from other body sites, were omitted.

Screening and Data Extraction

All retrieved studies were imported into Zotero (Corporation for Digital Scholarship, Virginia, USA) reference management software to manage and remove duplicates. Titles and abstracts were screened for relevance, with a focus on studies directly linking gut microbiota to chronic disease pathogenesis or therapeutic outcomes. Full-text articles were assessed for relevance to the study. Data on study design, sample size, key findings, microbial species, and microbiome-based interventions (probiotics, prebiotics, FMT, etc.) were extracted from relevant studies.

Review and Synthesis

The collected studies were reviewed and synthesized to identify common themes, trends, and gaps in the literature. Special attention was given to mechanisms through which gut microbiota influences disease (e.g., SCFAs production, immune modulation, and inflammation). Microbial signatures associated with specific diseases, particularly T2DM, autoimmune conditions, and cancer, were also highlighted.

Therapeutic Implications, Including Microbiome-Based Interventions

By using the aforementioned literature search strategy, we aimed to provide a comprehensive overview of the current state of knowledge regarding the role of the gut microbiota in chronic diseases and highlight potential areas for future research. This was done using strategies developed through consulting team members and published literature on the subject of biomedical reviews using the Scale for the Assessment of Narrative Review Articles (SANRA) [10]. SANRA is a tool designed to evaluate the quality of narrative review articles in biomedical journals. The synthesized findings were critically evaluated, analysed, and then presented narratively. 

Ethical Considerations

Ethical approval was unnecessary as the study involved analyzing preexisting published data. This narrative review adheres to ethical principles and guidelines for conducting narrative studies. No primary data collection from human subjects was performed for this review; therefore, ethical approval was not required.

Historical perspective

Recent studies have demonstrated a link between GM composition and metabolic disorders. The recognition of the gut microbiota’s influence on health and disease has evolved significantly over the past century, from early speculation to the contemporary understanding of its critical role in various chronic conditions. Historically, the human gut microbiota was considered merely a passive collection of microorganisms that played a secondary role in digestion [11]. However, as research has progressed, the complexity and importance of the microbiome have become increasingly evident.

Early Observations (Pre-20th Century)

Although the concept of microorganisms in the human body dates back to the pioneering work of Antonie van Leeuwenhoek in the 17th century, who first described bacteria from his own mouth, the idea that these microbes might influence health was not widely acknowledged. In the late 19th and early 20th centuries, microbiologists such as Louis Pasteur and Robert Koch made significant contributions to understanding the role of pathogens in infectious diseases. However, the microbial communities residing in the gut were either largely ignored, considered insignificant, or were simply considered part of the digestive process [11-12].

The Microbiome Concept Emerges (Mid-20th Century)

It wasn't until the mid-20th century that microbiologists began to appreciate the gut as a complex ecosystem. Early studies of the mid-20th century focused on the human gastrointestinal tract. During this time, research was only focused on a limited number of bacterial species. Researchers were more focused on studying pathogens than commensals. There was more focus on theories that revolved around the role of the GM in digestion, and little thought was given to its wide impact on systemic health.

By the 1970s and 1980s, the focus of the scientific community shifted towards recognizing the diverse and dynamic microbial population harbored by the gut. With the advent of more day techniques, researchers were able to culture a broader range of microbes, including the ones that were not culturable previously [11-12].

Gut Microbiota and Disease (Late 20th Century)

By the end of the 20th century, significant advances were made in the field of molecular biology. Polymerase chain reaction and deoxyribonucleic acid (DNA) sequencing aided in a deeper understanding of the microbiome’s makeup. The Human Microbiome Project was started in 2007. This project was a landmark achievement. The United States National Institutes of Health funded this project. Scientists targeted to map the microbiome on and within the human body. The project revealed a detailed look at the diverse microbiota and the potential impacts it may have on health [13]. Scientists began to suggest a potential link between dysbiosis and the onset of various chronic medical conditions. T2DM, IBD, and obesity were found to be associated with dysbiosis. 

Role of the Microbiota in Chronic Disease (21st Century)

The beginning of the 21st century was a time of dramatic shift in the way researchers thought about the microbiome. Particularly, the role of the GM in chronic diseases. Research was more focused on the effects of the GM on metabolic processes, neurological health, and immunity. It was during this period that the GM was identified as a key regulator of health. Researchers found evidence linking gut dysbiosis to diseases such as cancer, diabetes, and autoimmune disorders [14].

The 2010s brought a surge of interest in cancer research and the microbiome’s role in carcinogenesis. Research suggested that the GM could either stimulate or impede tumor development in various cancers. Research has found that certain bacterial species, such as *Fusobacterium nucleatum*, have been implicated in the development of gastrointestinal cancers, particularly colorectal cancer. Thus, suggesting that targeting the gut microbiome could be a viable approach in both cancer prevention and therapy. [15-16].

Gut microbiota and diabetes

Recent studies have suggested a link between the composition of the GM and metabolic conditions like T2DM. Certain bacterial species have been found to cause glucose intolerance and insulin resistance. A weakened gut barrier as a result of decreased butyrate-producing bacterial population can cause systemic inflammation and may consequently contribute to developing T2DM. Altering the GM with the use of probiotics, prebiotics, and dietary interventions has led to better metabolic outcomes in diabetics [17-18].

Gut Microbiota Composition in Diabetes

A balanced and diverse microbiota is crucial for maintaining metabolic health. Dysbiosis has been involved in the development of metabolic disorders, such as diabetes. Studies have suggested that the GM is significantly altered in T2DM. There is reduced microbial diversity and an overexpression of inflammation-promoting bacteria [17-18].

A consistent finding in diabetic patients is the microbial imbalance between Bacteroides and Firmicutes species, which contributes to insulin resistance. *Faecalibacterium prausnitzii* and *Roseburia hominis *are known for their beneficial effects on gut health and metabolism. These are often found to be reduced in individuals with T2DM [18].

Mechanisms Linking Gut Microbiota to Insulin Resistance

The GM influences insulin sensitivity and glucose metabolism through several mechanisms. One of the most important pathways is the production of SCFAs. These are produced as a result of dietary fiber fermentation by gut bacteria. SCFAs, particularly acetate, butyrate, and propionate, have been shown to improve insulin sensitivity by regulating glucose homeostasis. They also enhance the function of insulin-producing pancreatic cells. Release of regulatory hormones like glucagon-like peptide 1 is also influenced by SCFAs [19-20].

On the contrary, the GM can also affect insulin resistance. Dysbiosis has been linked to cause endotoxaemia (release of lipopolysaccharides into the bloodstream). This triggers a chronic but low-grade inflammatory response, leading to insulin resistance. As a result of this inflammatory response, the nuclear factor kappa B (NF-κB) pathway is activated. This impairs insulin signaling and aggravates metabolic dysfunction [20]. Additionally, studies have found that dysbiosis can promote obesity. Insulin resistance and T2DM are the two major sequelae of obesity [21].

Gut Microbiota as a Therapeutic Target in Diabetes

Given the growing body of evidence linking the GM to diabetes, researchers have increasingly focused on microbiome-based therapeutic strategies for managing and preventing T2DM. The potential to modulate the GM through dietary interventions, probiotics, prebiotics, and FMT offers promising new avenues for diabetes treatment (Table 1) [21-27].

**Table 1:** Gut microbiota based therapeutic strategies for diabetes management Dietary interventions, probiotics, prebiotics, and FMT are emerging strategies to modulate gut microbiota and improve metabolic health. Fiber-rich diets promote the growth of SCFA-producing bacteria, which enhance insulin sensitivity. Probiotics and prebiotics support microbial diversity and reduce inflammation. FMT offers potential for restoring microbial balance and improving glucose metabolism, though its long-term effects require further investigation. FMT: fecal microbiota transplantation; SCFA: short-chain fatty acid; References: [21-27]

Therapeutic Approach	Mechanism/Effect	Examples
Dietary Interventions	Promotes microbial diversity and SCFA production	High fiber diets, whole grains, vegetables
Probiotics and Prebiotics	Improve insulin sensitivity and reduce inflammation	Lactobacillus, Bifidobacterium, inulin
FMT	Restores microbial balance, enhances glucose metabolism	FMT from healthy donor (experimental)

Gut microbiota and autoimmune diseases

The role of the GM has been found to be critical in regulating the immune system. Dysbiosis has been involved in various autoimmune disorders, such as IBD, RA, and MS. Changes in microbial diversity can disturb the immune homeostasis. This leads to abnormal immune responses to self-antigens. Therefore, therapeutic approaches targeted at fixing microbial balance are being studied as potential treatments in such disorders. These include the use of FMT and targeted probiotic therapies [28]. Recently published literature has found a complex interaction that exists between immune modulation, GM, and autoimmunity. Thus, suggesting that the gut has a vital role in alleviating or worsening autoimmune responses [29-30].

Immune System and Gut Microbiota Interactions

The gut harbors a vast and heterogeneous microbial ecosystem. The host immune system is in constant interaction with this ecosystem. Nearly 75% of the body's immune cells reside in the gastrointestinal tract. Particularly, in structures like gut-associated lymphoid tissue and Peyer’s patches, thus making the GM a key figure in immune response regulation. The microbiota promotes immune tolerance under normal conditions. This helps prevent aberrant immune reactions that may lead to autoimmune diseases. On the other hand, alterations in the GM can impair this intricate balance, which leads to immune dysregulation and, thereby, the onset of autoimmune responses [31].

The GM influences immune function through several mechanisms. One of the most important pathways is the production of SCFAs. SCFAs, particularly acetate, butyrate, and propionate, have been shown to regulate immune homeostasis by promoting the differentiation of regulatory T cells and the suppression of proinflammatory T helper 17 (Th17) cells activation. These mechanisms help prevent aberrant immune responses targeting the body’s own tissues that lead to autoimmune pathology [32]. When the use of antibiotics, diet, or infections causes dysbiosis, the GM may promote inflammation and immune activation.

Gut Microbiota in Rheumatoid Arthritis, Multiple Sclerosis, and Inflammatory Bowel Disease

The connection between GM and RA, MS, and IBD has been a subject of intense investigation in recent years. Table 2 outlines the key microbial changes, associated immune responses, and therapeutic implications observed in these three autoimmune conditions.

**Table 2:** Summary of gut microbiota alterations and their immunological implications in autoimmune diseases Notable findings include the disproportionate abundance of *Prevotella copri *in RA, reduced abundance of *Faecalibacterium prausnitzii *in MS, and dysbiosis marked by a decrease in butyrate-producing bacteria in IBD. Each condition shows distinct patterns of microbial imbalance linked to systemic inflammation, autoimmunity, and disease progression. The table also highlights current microbiota-targeted therapeutic strategies, including probiotics, prebiotics, and dietary interventions aimed at restoring microbial balance and reducing disease severity. RA: rheumatoid arthritis; MS: multiple sclerosis; CNS: central nervous system; SCFA: short-chain fatty acid; IBD: inflammatory bowel disease; ↓: decreased bacterial population; ↑: increases bacterial population Reference: [33-40]

Autoimmune Disease	Key Microbial Findings	Mechanisms Involved	Notable Microbial Species	Potential Therapeutic Strategies
RA [33-34]	Dysbiosis with increased proinflammatory bacteria and reduced beneficial microbes	Gut dysbiosis may trigger systemic inflammation and autoantibody production; activation of T and B cells	Prevotella copri (pro-inflammatory)	Probiotics, prebiotics, dietary interventions to reduce inflammation and restore microbial balance
MS [35-38]	Reduced microbial diversity and altered abundance of specific taxa	Dysbiosis promotes neuroinflammation and CNS autoimmunity via immune modulation	↓ *Faecalibacterium prausnitzii* (butyrate producer, anti-inflammatory)	Probiotics, dietary changes to increase beneficial microbes, and SCFA production
IBD [39-40]	Decreased *Firmicutes* and *Bacteroidetes*; increased harmful/pathogenic bacteria	Immune hyper-responsiveness to altered microbiota leads to chronic intestinal inflammation	↑ *Escherichia coli*, *Enterococcus faecalis*, Adherent Invasive *E. coli*; ↓ butyrate producers	Probiotics, prebiotics, SCFA supplementation, and microbiota-targeted diets to restore gut integrity

Gut microbiota and cancer

The role of GM in cancer pathogenesis is very complex. Research has found bacterial populations that produce metabolites that promote carcinogenesis. While some have anti-cancer or protective effects, some bacteria break down dietary products into cancer-causing compounds, while others produce SCFAs with anti-inflammatory and anti-cancer properties. Therefore, preventive and therapeutic approaches targeted at fixing microbial balance are being studied as potential treatments in such disorders [41-42]. The role of the GM as a central player in the development, progression, and prevention of cancer has been the subject of research, particularly its role in gastrointestinal cancers [43].

Role of the Gut Microbiota in Carcinogenesis

The gut harbors a vast and heterogeneous microbial ecosystem. The host immune system is in constant interaction with this ecosystem. Recent findings suggest that dysbiosis can promote carcinogenesis through multiple pathways. These include the production of harmful metabolites, chronic inflammation, and alteration of host immunity [43]. Chronic inflammation is one of the primary mechanisms that contribute to the development of cancer.

Certain pathogenic microbes within the gut can trigger an inflammatory response that damages DNA and tissues. This creates an environment favourable for cancer development. For instance, *Fusobacterium nucleatum*, a normally found gut bacterium, has been shown to promote colorectal cancer by stimulating inflammatory pathways. This state of chronic inflammation can lead to carcinogenesis via DNA mutations and epithelial to mesenchymal transition [44].

Gut Microbiota in Colorectal, Gastric, Esophageal, and Liver Cancers

An overview of the gut microbiota’s involvement in the pathogenesis of colorectal, gastric, esophageal, and liver cancers is provided in Table 3. Together, these findings underscore the microbiota's multifaceted role in gastrointestinal cancer risk and progression.

**Table 3:** Gut microbiota-associated alterations and their roles in gastrointestinal cancers In CRC, pro-carcinogenic species such as *Fusobacterium nucleatum*, Enterotoxigenic *Bacteroides fragilis *(ETBF), and *Escherichia coli *promote tumorigenesis through inflammation, immune modulation, and DNA damage, while butyrate-producing bacteria exhibit protective effects via anti-inflammatory and pro-apoptotic mechanisms. In gastric cancer, *Helicobacter pylori *infection and accompanying microbial shifts contribute to chronic inflammation and carcinogenesis. Dysbiosis in esophageal cancer is associated with microbial imbalance and metabolite-induced epithelial changes. For liver cancer, microbial products transported via the portal vein influence hepatic inflammation and fibrosis, linking gut microbiota composition to hepatocellular carcinoma development. CRC: colorectal cancer; DNA: deoxyribonucleic acid; SCFA: short-chain fatty acid; NAFLD: nonalcoholic fatty liver disease; LPS: lipopolysaccharides References: [43-51]

Cancer Type	Key Microbial Involvement	Mechanisms	Notable Microbes/Metabolites	Potential Implications
CRC	Dysbiosis promotes carcinogenesis; imbalance between pathogenic and beneficial microbes [43]	Pathogens induce inflammation, DNA damage, immune evasion; SCFAs suppress tumorigenesis [19,20]	*Fusobacterium nucleatum*, ETBF, *Escherichia coli* (pro-carcinogenic); SCFA-producing bacteria like *Faecalibacterium prausnitzii* (protective) [43,45]	Targeting microbiota to reduce inflammation and enhance SCFA production
Gastric Cancer [46-47]	*Helicobacter pylori* infection alters gastric microbiota	Chronic inflammation, DNA damage, epithelial hyperplasia	*Helicobacter pylori*; altered gastric microbiota profiles	Microbiota signatures may indicate susceptibility or protection
Esophageal Cancer [48-49]	Dysbiosis with increased pathogenic bacteria and reduced beneficial taxa	Microbial metabolites (e.g., bile acids, SCFAs) influence carcinogenesis	Pathogenic bacteria; bile acids; SCFAs	Microbiota-driven modulation of immune and epithelial responses
Liver Cancer [50-51]	Gut-liver axis via portal circulation of microbial products and metabolites	Dysbiosis promotes NAFLD, cirrhosis, inflammation, and fibrosis	Microbial LPS, ethanol, SCFAs, dysbiosis-related species	Targeting microbiota may prevent progression to hepatocellular carcinoma

Current understanding and future directions

Today, the GM is recognised not only as a critical player in digestion but also as a powerful regulator of metabolic, immune [52], and even neurological functions. The concept of dysbiosis has shifted from being a secondary factor to a central one, with researchers focusing on understanding the specific microbial populations and their functional roles in chronic disease pathogenesis, and on drugs' efficacy and their side effects [53]. The ability to influence the microbiome through interventions such as prebiotics, probiotics, and FMT is rapidly advancing. This offers promising therapeutic opportunities [24-27].

The relationship between gut microbiota and chronic diseases such as diabetes, autoimmunity, and cancer continues to be a major area of interest. The goal is to translate these insights into novel, microbiome-based treatments. The future research holds promise for developing new therapeutic approaches that control the GM's power to both combat disease and promote health.

Limitations

There are a few limitations to this narrative review. Firstly, there was limited data available on the subject. Secondly, we only included articles that were published in the English language. Studies that were published in other languages were not included in the literature review for this study. It is recommended that more translational studies be conducted to explore the role of gut microbiota in cancer, diabetes, and autoimmune conditions.

## Conclusions

The GM represents an intricate, dynamic, and complex ecosystem. It plays a key role in not only maintaining the host's health but also in influencing the development and progression of the aforementioned three conditions. Recent advances have validated that dysbiosis may lead to immune dysregulation, systemic inflammation, and metabolic disturbances. Altering the composition and function of the GM offers an opportunity for innovative therapeutic strategies targeted at disease management and prevention. FMT, probiotics, prebiotics, and dietary modifications are being explored for their potential to restore microbial balance and better clinical outcomes. Further research and advancements may carry the potential to modify the landscape of chronic disease management. This can pave the way for a more holistic and precise approach to healthcare.
